# The Ovarian Transcriptome of Reproductively Aged Multiparous Mice: Candidate Genes for Ovarian Cancer Protection †

**DOI:** 10.3390/biom10010113

**Published:** 2020-01-09

**Authors:** Ulises Urzúa, Carlos Chacón, Maximiliano Norambuena, Luis Lizama, Sebastián Sarmiento, Esther Asaki, John I Powell, Sandra Ampuero

**Affiliations:** 1 Laboratorio de Genómica Aplicada, Departamento de Oncología Básico-Clínica, Facultad de Medicina, Universidad de Chile, Santiago 8380453, Chile; 2 Programa de Virología, ICBM, Facultad de Medicina, Universidad de Chile, Santiago 8380453, Chile; 3 Center for Information Technology, National Institutes of Health, Bethesda, MD 20892, USA

**Keywords:** age, parity, ovary, transcriptome, follicle, inflammation, mouse model

## Abstract

In middle-aged women, the decline of ovarian follicle reserve below a critical threshold marks menopause, leading to hormonal, inflammatory, and metabolic changes linked to disease. The highest incidence and mortality of sporadic ovarian cancer (OC) occur at post-menopause, while OC risk is reduced by full-term pregnancies during former fertile life. Herein, we investigate how parity history modulates the ovarian transcriptome related to such declining follicle pool and systemic inflammation in reproductively-aged mice. Female C57BL/6 mice were housed under multiparous and virgin (nulliparous) breeding regimens from adulthood until estropause. The ovaries were then subjected to follicle count and transcriptional profiling, while a cytokine panel was determined in the sera. As expected, the follicle number was markedly decreased just by aging. Importantly, a significantly higher count of primordial and total follicles was observed in aged multiparous relative to aged virgin ovaries. Consistently, among the 65 genes of higher expression in aged multiparous ovaries, 27 showed a follicle count-like pattern, 21 had traceable evidence of roles in follicular/oocyte homeostasis, and 7 were transforming-growth factor beta (TGF-β)/bone morphogenetic protein (BMP) superfamily members. The remaining genes were enriched in cell chemotaxis and innate-immunity, and resembled the profiles of circulating CXCL1, CXCL2, CXCL5, CSF3, and CCL3, chemokines detected at higher levels in aged multiparous mice. We conclude that multiparity during reproductive life promotes the retention of follicle remnants while improving local (ovarian) and systemic immune-innate surveillance in aged female mice. These findings could underlie the mechanisms by which pregnancy promotes the long-term reduced OC risk observed at post-menopause.

## 1. Introduction

Aging is characterized by a wide range of cumulative damage that affects tissue homeostasis, thus predisposing to disease initiation. Cell signaling and metabolism, antioxidant defense, epigenetic status, intercellular communication, and genome stability, among other functions, become significantly impaired [1]. In the mammalian ovary, such age-related decline is largely governed by a sustained decrease of the quantity and quality of the oocyte pool enclosed in the ovarian follicles at different stages of maturation. Follicle depletion in women leads to a dysregulation of the hypothalamic–pituitary–gonadal axis culminating at menopause, a hallmark of women’s reproduction characterized by major systemic endocrine, metabolic, and inflammatory changes [2].

Increased rates of chronic diseases, including several types of cancer, are observed in menopause. The declining follicle number has been recently proposed to play a role in ovarian cancer (OC) etiology by promoting a pre-neoplastic phenotype of the ovarian surface epithelium (OSE) [3,4,5], a cell layer claimed to be the origin of the most frequent OC variant, the epithelial OC [6]. According to this idea, once the ovary is depleted of follicles, OSE would be released of a proliferative blockade maintained by a paracrine factor from follicular granulosa cells [7]. An additional genetic lesion in a cancer driver gene would be necessary in order to initiate ovarian carcinogenesis [8]. The post-menopausal ovary develops several age-related morphological alterations, such as stromal fibrosis, vascular remodeling, OSE invaginations, and epithelial inclusion cysts, the latter regarded as pre-neoplastic lesions [9,10]. Consistent with these changes, OC incidence and mortality steadily increase with age, reaching a maximum during the peri-menopause and early post-menopause periods [11]. Most of the observed menopause manifestations synergize with the age-dependent systemic low-grade inflammation underlying the development of chronic diseases, including OC [12].

Equally important, the observed OC risk at menopause is significantly decreased by reproductive history during the previous fertile life. Conditions characterized by a reduced number of ovulatory events, namely parity and the use of oral contraceptives, have been recognized to reduce OC risk in various populations [11]. The underlying basis of this low-risk effect would be a decreased tear-repair, ovulatory damage to the OSE during fertile life [6]. However, despite the proposed role of follicle depletion in OC pathogenesis [3,4,5], much less attention has been paid to how parity and oral contraceptives might affect the number of follicles remaining at menopause, which is around 1000 non-growing follicles in women [13]. To date, just a single study has linked parity to a higher ovarian reserve in women of reproductive age [14].

Mouse models have been used to study OC risk factors, including obesity [15], inflammation [16], age [8], and genetic inheritance [17], among others. Continuing the recent work on peritoneal tumor spread and systemic inflammation in an aged syngeneic female C57BL/6 mouse model of OC maintained in multiparous and nulliparous regimens [18], here, we studied the effect of parity history on the ovarian transcriptome in intact, uninduced animals. Increased transcript levels of several markers of oocytes and follicles concomitant with significantly higher residual follicle counts were observed in multiparous ovaries. A second gene-expression pattern overexpressed in aged multiparous ovaries was correlated with circulating chemokines levels, and revealed a functional enrichment in leukocyte chemotaxis and the regulation of innate immunity and inflammation. The results are discussed regarding how such higher residual follicular remnants confer an improved immunosurveillance capacity to the aged multiparous ovary, thereby reducing the risk of neoplasia initiation in the ovary.

## 2. Materials and Methods

### 2.1. Animals, Study Scheme, and Sample Collection

**Figure 1:**
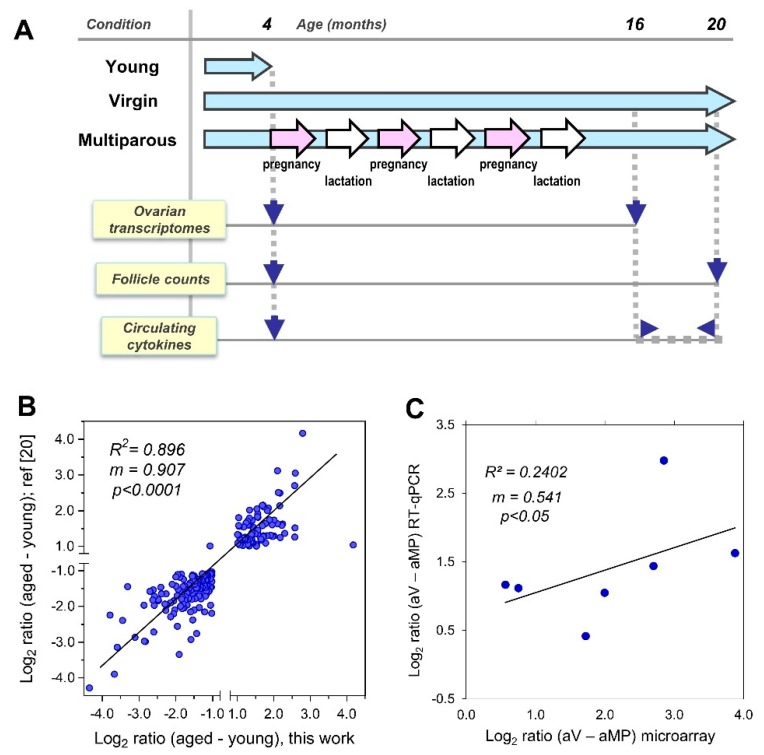
Study flowchart and platform validations: (**A**) Female C57BL/6 mice were housed from 3 to 20 months in virgin (nulliparous) and multiparous conditions (*n* = 70 per group). A group of young adult, four-month-old virgin mice (*n* = 20) was used as a reference. Transcriptomic profiling was done in young (*n* = 6), 17-month-old virgin (*n* = 5) and 17-month-old multiparous mice (*n* = 6). The follicle count was performed in young (*n* = 3), 20-month-old virgin (*n* = 16) and 20-month-old multiparous mice (*n* = 18). Serum cytokines were measured at the diestrous stage in adult young, four-month-old mice (*n* = 6) and in randomly chosen, 16–20 month-old female mice of both virgin and multiparous groups (*n* = 4 per group/month). The time scale shown is not proportional. (**B**) Scatter plot for a 225 genes subset comparing our expression profiling data in young (*n* = 6) versus aged ovaries, irrespective of parity (*n* = 11), with that obtained with the NIA-22k platform (*n* = 2 per group) [20]. Data points between −1.0 and 1.0 log_2_ range were omitted. (**C**) Scatter plot depicting RT-qPCR and microarray results for *Foxo1*, *Fst, Fshr, Alas2*, *Snca*, *Hba-a1,* and *Hbb-bt* transcripts in the log_2_ scale for both platforms, so as to compare the transcription of aged-virgin versus aged multiparous ovaries; *n* = 3 per group in RT-pPCR assays, *n* = 5 aged virgin, and *n* = 6 aged multiparous in microarray data. The squared correlation, slope, and *p*-values are shown within the plot.

This study with female C57BL/6 mice was approved by the Bioethics Committee, Faculty of Medicine, University of Chile (CBA # 0536 FMUCH). The care and monitoring of the two experimental groups, virgin and multiparous, have recently been described in detail [18]. Figure 1A depicts the experimental design for the present report. Briefly, a subset of 12 animals (*n* = 6 per group) around 16-months old was euthanized to collect ovaries for RNA extraction and subsequent gene expression profiling with microarrays (see below). At 20 months old, another subset of 34 mice (*n* = 16 virgin, *n* = 18 multiparous) was euthanized to dissect ovaries for follicle count. Sera were obtained from a third subset (*n* = 8 per group) from the blood samples collected twice per month from 17–20 months old. Both ovarian tissue and serum samples from four-month-old mice (*n* = 6 per group) were used as a reference.

### 2.2. Count of Ovarian Follicles

The dissected ovary pairs were fixed in 1% *p*-formaldehyde and embedded in paraffin. The largest longitudinal sections, 5-μm thick, were stained with haematoxylin-eosin (HE) and mounted in Entellan^®^ (Merck, Darmstadt, Germany). Primordial, primary, preantral, secondary, and Graafian follicles were identified according to the morphological criteria of Griffin et al. [19], with one single modification, namely: both the incipient and small antral follicles described by these authors are named here as secondary follicles. The follicle counts per sample consisted of the sum of each follicle type in the largest single longitudinal section containing the ovary pair. Multiparous animals had at least two litters (range 2–7, mean 3.5). The mean ages were 21.71 and 20.94 months old for virgin and multiparous mice, respectively.

### 2.3. Microarray Profiling of Mouse Ovaries

One-channel bead-DNA microarrays (MouseRef-8 v2.0, Illumina, Foster City, CA, USA) composed of 25,697 probes (80-mer) covering 17,640 unique coding genes were used. Each probe was represented 25–45 instances in a single microarray for a total of over 900,000 probes per microarray. A set of 17 microarrays comprised young (*n* = 6), aged virgin (*n* = 5), and aged multiparous (*n* = 6) ovary samples. The total ovarian RNA was isolated with an All-Prep kit (Qiagen, Hilden, Germany, GmbH), treated with Turbo-DNAse (Ambion, Austin, TX, USA), precipitated with sodium acetate plus absolute ethanol, and stored at −80 °C until use. The RNA was quantified by UV spectrophotometry (A260/A280 nm) and verified for integrity with an Agilent 2100 Bioanalyzer (Agilent Tech, Palo Alto, CA, USA). All of the RNA samples had RNA integrity (RIN) values > 7.5. Transcriptional profiling was performed by the service provider Macrogen Inc. (Seoul, Korea). Further experimental details are given in Supplementary File 2.

### 2.4. Analysis of Transcriptomic Data

The scanned images were analyzed with the Illumina Genome Studio v2011.1, gene expression module v1.9.0. The raw data was deposited at the NIH’s-National Cancer Institute microarray database (http://nciarray.nci.nih.gov). After quantile normalization, the differentially expressed genes (DEGs) were determined by limma tests with FDR adj *p*-values <0.05 and log_2_ fold-change (FC) >±1.0 for the young-aged comparison. The cut-offs for the aged virgin-aged multiparous comparison were FDR adj *p*-values <0.10 and log_2_ FC >±0.8. Quality threshold clustering (QTC), heatmaps, and centroid plots were done with MeV (http://mev.tm4.org/). Gene ontology (GO) enrichment was done with Visual Annotation Display(VLAD) v1.5.1 (http://proto.informatics.jax.org/prototypes/vlad/) and WebGestalt (http://www.webgestalt.org/). Gene Set Enrichment Analysis (GSEA) was done at (http://software.broadinstitute.org/gsea/index.jsp). Protein–protein interaction networks were analyzed with STRING-v11.0 (https://string-db.org/). Mouse immune cell gene expression data were extracted from the Ref-DIC database (http://refdic.rcai.riken.jp/profile.cgi).

### 2.5. Determination of Circulating Chemokines

Mouse cytokines CXCL1, CXCL2, CXCL5, CCL3, and CSF3 were quantified in serum using the multiplex, magnetic bead-based assay MCYTMAG-70K-PX32 panel from Milliplex^®^ (Merck, Kenilworth, NJ, USA), following instructions by the manufacturer, as previously described [18]. Before running the assay, samples were randomized, and their identities were made blind to the operators. Additional experimental details are provided in Supplementary File 2.

### 2.6. Quantitative RT-PCR Assays

One µg of DNAse-treated RNA was reverse-transcribed with 20 U of M-MLV (Promega, Madison, WI, USA), oligo-dT plus random hexamer primers for 1 h at 42 °C, and then inactivated for 10 min at 70 °C. Quantitative PCR was performed by combining an aliquot of the resultant cDNA with 1 µM final of each forward and reverse primers and Sensimix Sybr HI-ROX reagent (Bioline, London, UK). Primer pairs were designed with the Primer3-Plus software [21]. Three samples per experimental condition and triplicate technical assays per sample were performed in a RotorGene™ thermocycler (Corbett Life Sciences, Sydney, Australia). Further details in Supplementary File 2.

### 2.7. Statistics

The follicle counts and cytokine levels were plotted as mean ± standard error of the mean. The data did not follow a normal distribution, and were compared as non-paired data between the aged groups by using Mann–Whitney tests (non-parametric). Significance was set at *p* < 0.05. The analysis was performed with the GraphPad Prism 5.0 software.

## 3. Results

### 3.1. Study Scheme and Validation of Platforms

We recently described the reproductive records, circulating gonadotropin levels, systemic inflammatory status, and intraperitoneal tumor spread in estropausal C57BL6 female mice, as well as the differential accumulation of lipofuscin and hemosiderin in the ovaries of these animals according to their divergent parity history [18,22]. In extending these studies, here, we explore how pregnancy impacts the post-reproductive ovary, with a focus on the residual ovarian reserve and the follicular and cell immunity-related transcriptome (Figure 1A). Firstly, to assess the reliability of this Illumina platform, we compared expression ratios of over 200 differentially expressed genes between aged and young ovaries in the <−1.0 and >1.0 log_2_ ratio range with a related previous study [20], in which the NIA Mouse 44 K v2.1 and v2.2 platforms were used (Agilent, Santa Clara, CA, USA). As shown in Figure 1B, we observed a significant positive correlation with a slope close to 1.0, meaning that both the magnitude and direction of the changes were equivalent between the platforms. Also, the levels of seven selected transcripts were confirmed using RT-qPCR, for which the cycling conditions and primer pairs sequences are described in Supplementary File 2. The comparison of the transcript levels between the aged virgin/aged multiparous ovaries measured by both the MouseRef-8 v2.0 platform and the RT-qPCR assays also revealed a significant positive correlation. The observed slope suggests that the transcript levels were in a different range of magnitudes for each platform (Figure 1C).

### 3.2. Remnant Follicles in Aged Mouse Ovaries of Divergent Parity History

**Figure 2:**
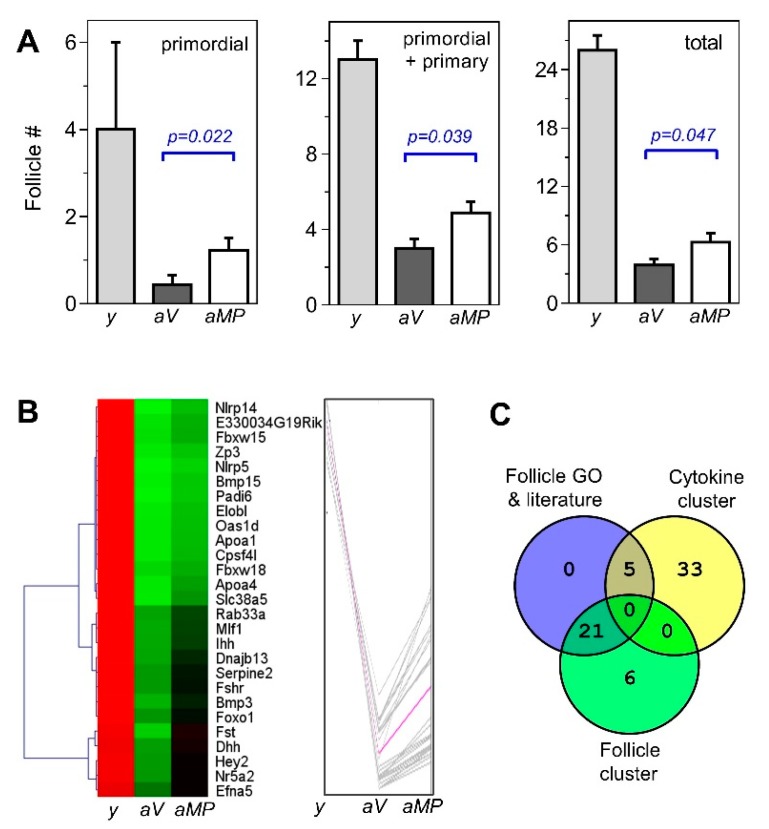
Ovarian follicle counts and cognate ovarian gene expression. (**A**) Mean (± standard error of the mean (SEM)) primordial, primordial plus primary, and total follicle counts in young (*y*), aged virgin (*aV*), and aged multiparous (*aMP*) ovaries; *p*-values are indicated. (**B**) Heatmap of the follicular-like signature (27 differentially expressed genes (DEGs); rows) in ovaries of the three experimental groups (*y*, *aV*, and *aMP*) showing mean absolute levels of quantile normalized log_2_ scale values. Quality threshold clustering (QTC) clustering was done with MeV (http://mev.tm4.org/) using the Euclidean distance and 0.42 diameter with prior gene/row adjustment divided by root mean square (RMS). The 27-genes cluster was part of the 65 DEGs of a higher expression in the multiparous ovaries (see text). (**C**) Venn diagram of the 26 follicle associated genes “Follicle GO and literature” according to gene ontology (GO) (Table 2) and literature analysis (Table 3), the 27 genes showing the follicular-like signature, “Follicle cluster”, and the 38 genes of the “Cytokine cluster” (see Section 3.6).

The follicles at various stages were identified and counted in the ovaries of the young and the two aged conditions. Based on the mean follicle counts, the ovaries of both aged groups contained significantly lower total follicle counts compared with the young ovaries, as expected, because of the age-dependent ovarian decline (Figure 2A, rightmost chart).

This result is consistent with the increased levels of gonadotropins and estrous cycle lengthening observed by the 16–20 months-old in this animal cohort [18]. Interestingly, follicle depletion was less pronounced in the ovaries of the aged multiparous than in those of the aged virgin mice. The mean counts of the primordial, primordial plus primary, and total follicles were significantly higher in the aged multiparous relative to aged virgin ovaries (Figure 2A). A simple calculation of [aged multiparous/aged virgin] follicles ratios using mean follicle values, resulted in 2.8 and 1.6 for the primordial and total follicles respectively, which suggests a relative enrichment of primordial follicles in aged multiparous with respect to aged virgin ovaries. This finding agreed with a higher co-expression of the genes involved in the maintenance of the primordial follicle pool or preventing their cyclic recruitment in aged multiparous relative to aged virgin ovaries (see Section 3.4.1 and Section 4.1).

### 3.3. The Differential Mouse Ovarian Transcriptome: Age and Parity Comparisons

To study the effect of age and parity history on the mouse ovarian transcriptome, limma tests were conducted for the following two-group comparisons: First, young ovaries (*n* = 6) versus aged ovaries irrespective of parity status (*n* = 5 + 6 = 11) resulted in 905 DEGs (adj *p* < 0.05 and ±1.0 log_2_ FC). This comparison allowed us to interpret parity differences below (Section 3.4) over an aged “baseline” gene transcription. This gene list was decomposed in 489 up-regulated and 416 down-regulated genes in aged relative to young ovaries. To our knowledge, transcriptomic studies of post-reproductive versus fertile-age human ovaries have not been reported so far. Just two proteomic studies compared pre- versus post-menopausal ovaries in small (*n* = 3–4 per group) sample sizes (Table 1). Moreover, neither a single-gene nor transcriptomic parity-dependent gene expression study has been previously addressed in the human postmenopausal ovary.

**Figure 3:**
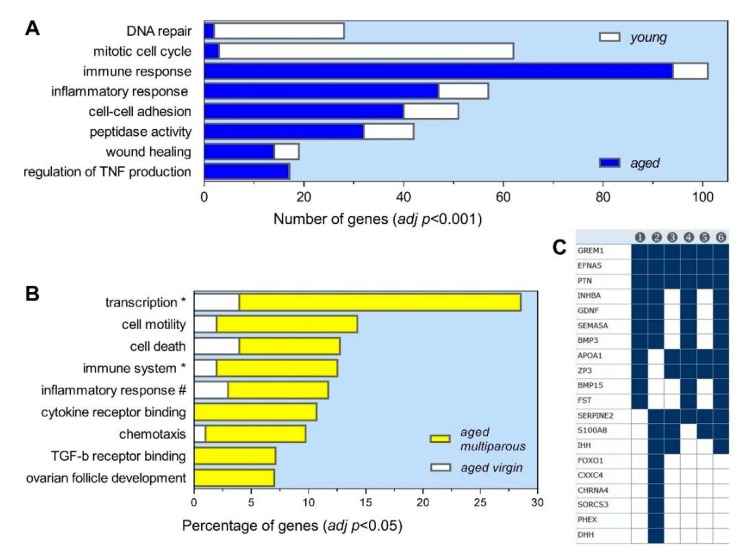
Gene ontology (GO) profile of differentially expressed genes in aged ovaries. Analysis of DEGs between (**A**) aged versus young, and (**B**) aged virgin versus aged multiparous ovaries after limma tests, as described in Methods. The aged/young test resulted in a similar number of up- and down-regulated genes. As the aged virgin/aged multiparous comparison resulted in unbalanced up- and down-regulated gene numbers (65 versus 112), the GO enrichment was converted into percentages. The VLAD tool was run with official *M musculus* gene symbols (adj *p* < 0.05; gene function annotation from Mammalian Genome Informatics (MGI)) with default parameters. Abbreviated GO terms: (*) “positive regulation of”; (#) “regulation of”. (**C**) GSEA analysis of the genes upregulated in multiparous ovary using the C5 (GO) collection. Column headings are (1) receptor regulator activity (11 genes), (2) cell–cell signaling (16 genes), (3) extracellular matrix (10 genes), (4) molecular function regulator (16 genes), (5) collagen containing extracellular matrix (9 genes), and (6) signaling receptor binding (15 genes). All the terms met an FDR *q*-value < 4.1 × 10^−5^.

The gene ontology (GO) analysis indicated that, regardless of parity history, aged ovaries expressed a minimal repertoire of mitotic, cell cycle, and DNA repair genes, but a high content of genes implicated in inflammatory/immune responses, cell adhesion, TNF production, peptidase activity, and wound healing (Figure 3A). These functions agreed to a large extent with those described by Sharov et al. [20], and essentially reflect a typical senescent status with a low proliferative activity, increased tissue remodeling, and innate immune cross-talk, leading to chronic low-grade tissue inflammation known as the senescence-associated secretory phenotype (SASP) [23]. High expression levels of over two-dozen distinct cytokines, interleukins, their respective receptors, and interferon-related genes, in addition to typical macrophage, neutrophil, lymphocyte, and mast cell markers were detected in aged mouse ovaries (not shown). These included F4/80 (*Adgre1*), CD68, CD86, class II H2 complex (*H2-Aa*, *Ab1*, *DMa*, *DMb1*, and *Eb1*), Ly6 complex (*a, d,* and *e loci*), CD3 complex (*Cd3e, d,* and *g*), CD44, and proteases (*Cpa3* and *Cma1*). Furthermore, all of the GO terms describing the activation of the leukocyte, lymphocyte, macrophage, neutrophil, and mast cells were significantly enriched in the aged relative to young ovaries. The same trend was observed for the terms of “phagocytosis”, “lysosome”, “cell surface receptors”, and “cytokine-mediated signaling”, which are closely related to the SASP. Consistent with this local ovarian inflammation associated with the reproductive decline, we previously showed an age-dependent increase of systemic levels of several cytokines in this mouse cohort, including TNF-alpha, IL1-beta, CCL2, IL-10, IL-5, and IL-4, among others [18].

The second comparison was done between aged ovaries only, and was based on their parity history, that is, virgin (*n* = 5) versus multiparous (*n* = 6). This resulted in 177 DEGs (adj *p* < 0.10; ±0.8 log_2_ FC), which were decomposed in 65 up-regulated and 112 down-regulated genes in aged multiparous respective to aged virgin ovaries.

### 3.4. Gene Ontology Profile of the Aged Multiparous Mouse Ovary

Given the known effect of past pregnancy on OC risk in menopause [11], the analysis in this report was focused on the 65 DEGs of a higher expression in aged multiparous with respect to aged virgin ovaries. As shown in Figure 3B and summarized in Table 2, a significant enrichment in GO terms related to four major interrelated themes was observed, namely: (i) follicle and oocyte homeostasis, (ii) cell immunity and inflammation, (iii) transcriptional regulation, and (iv) cell death. The GSEA analysis pointed to a modulation of receptor activities, cell–cell signaling, and cell–extracellular matrix relationships (Figure 3C). The genes not appearing in Table 2 were *Serpine2*, *Cxxc4*, *Chrna4*, *Sorcs3*, and *Phex*, all under cell–cell signaling; except for *Serpine2*, this subset demonstrated a cytokine-like pattern (see Section 3.6), thus reinforcing the relevance of cell immunity and inflammation in the present analysis.

#### 3.4.1. Follicle and Oocyte Homeostasis

*Bmp15*, *Fshr*, *Inhba*, and *Oas1d* are annotated under “ovarian follicle development”, while *Dhh*, *Ihh*, and *Zp3* are annotated under “germ cell development”. *Bmp3*, *Bmp15*, *Gdnf*, and *Inhba* also display a “TGF-beta receptor binding activity”, whereas *Bmp3*, *Bmp15*, *Grem1*, and *Inhba* are indexed under the “regulation of pathway-restricted SMAD protein phosphorylation”. TGF-β-related genes *Bmp15*, *Bmp3*, *Fst*, and *Grem1* are also annotated in GO under the cognate pathway “bone morphogenetic protein (BMP) signaling”, while *Fst* and *Grem1* are classified as TGF-β antagonists. The GSEA analysis analogous to that of Figure 3C identified six genes in the C2-GCP overlap that were down-regulated in female Smad1/Smad5 conditional mutants that developed metastatic granulosa cell tumors [24]. SMAD proteins are the major signal transducers downstream of TGF-beta. A different nine-gene overlap was found to be upregulated in the uterus of *Bmp2* knock-out mice [25], and this might be interpreted as equivalent to the inhibition of BMP signaling by *Fst* and *Grem1* antagonists. An additional follicle-related pathway enriched in the aged multiparous ovary was the “steroid metabolic process” comprising the genes *Hsd17b1*, *Apoa1*, *Apoa4*, and *Nr5a2*. Including the Hedgehog signaling members *Ihh* and *Dhh*, known to modulate steroid biosynthesis in various organs, including the ovaries [26], there were six steroidogenic genes overexpressed in the aged multiparous relative to the aged virgin ovary.

As shown in Table 2, 15 of the 65 genes with a higher expression in the aged multiparous ovaries were indexed in oocyte/follicle related terms, according to the GO database. We additionally performed a manual literature search for oocyte/follicle associated genes, and found 11 further genes with experimental support to be involved in this function (Table 3). Therefore, a total of 26 out of the 65 (i.e., 41.5%) genes overexpressed in the aged multiparous with respect to aged virgin ovaries had evidence of roles in oocyte/follicle homeostasis.

#### 3.4.2. Cell Immunity and Inflammation

Included here were the GO terms “regulation of inflammatory response” (*Apoa1*, *Nlrp14*, *Nlrp5*, *S100a8*, and *Zp3*), “positive regulation of immune system process” (*Fpr2*, *Ihh*, *Inhba*, *Masp1*, *Rftn2*, and *Zp3*), “chemotaxis” (*Apoa1*, *Efna5*, *Fpr2*, *S100a8,* and *Sema5a*), and “cell migration” (*Apoa1*, *Fgf13*, *Fpr2*, *Gdnf*, *Grem1*, *S100a8*, and *Sema5a*). Among these 12 genes, *Fpr2* and *S100a8* overlap in three of the four GO terms mentioned. *Fpr2* codes for a member of the G-protein coupled formyl peptide receptor family, whereas *S100a8* codes for calgranulin-A, which is Ca/Zn binding protein that heterodimerizes with the calgranulin-B isoform. Both proteins confer a chemoattractant capacity to neutrophils and monocytes (see discussion).

#### 3.4.3. Transcriptional Regulation

This function comprised the GO term “positive regulation of transcription, DNA-templated” (*Bmp15, Bmp3, Cdk5rap2, Dbp, Foxo1, Gdnf, Grem1, Hey2, Hoxd10, Hoxd9, Ihh, Inhba, Nr5a2,* and *Zp3*). Six of these 14 genes (*Dbp, Foxo1, Hey2, Hoxd10, Hoxd9*, and *Nr5a2*) are annotated under the term “transcription factor activity, sequence-specific DNA binding”.

#### 3.4.4. Cell Death

The relevant GO terms containing the genes of the three themes above described were “cell death” (Figure 2B) and the “positive regulation of apoptotic process” (not shown). These terms comprised the gene *Ptn* plus the other five genes already mentioned (*Foxo1*, *Inhba*, *Nlrp5*, *S100a8,* and *Serpina3g*).

### 3.5. Correlation of Follicle Counts with the Ovarian Transcriptome

**Figure 4:**
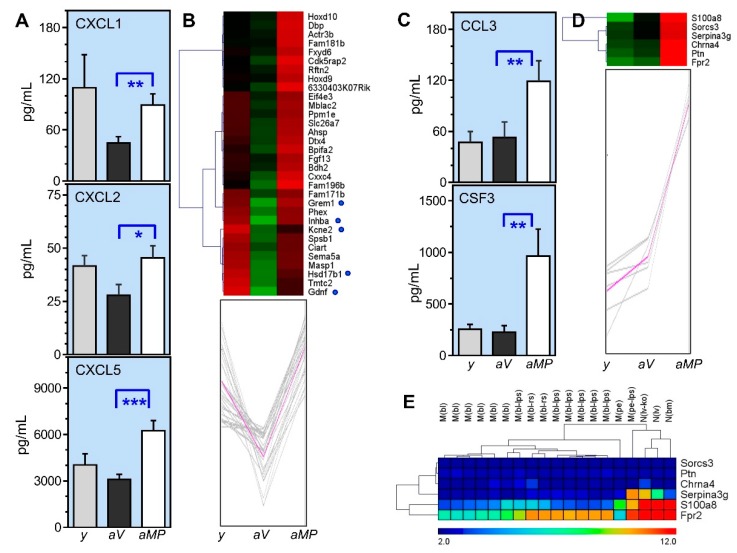
Parity-dependent circulating cytokines levels and related gene expression in aged ovaries: (**A**,**C**) Mean (±SEM) serum levels of the indicated cytokines in female young (*y*), aged virgin (*aV*), and aged multiparous (*aMP*) mice are shown with their respective 32-genes (**B**), and the six-genes (**D**) expression heatmaps and centroid clusters at the bottom obtained with same settings as that of Figure 2. Significant differences among the cytokine levels were determined after two-tailed Mann–Whitney tests, (*) *p* < 0.05, (**) *p* < 0.01, and (***) *p* < 0.001. Blue dots in the heatmap of (**B**) indicate genes with a cytokine-like pattern, but evidence of follicle function. Note that the sum of 27-genes (Figure 2B) and the 32 and 6 (38) genes in (**B**,**D**) complete the 65 DEGs of a higher expression in multiparous ovaries (see Section 3.3). The heatmap in (**E**) show the expression levels of the six-gene clusters in peritoneal and intact LPS-treated J774.1 macrophages (M label) and neutrophils (N label). Multiple samples correspond to different LPS doses and incubation times, as described in the Ref-DIC database (http://refdic.rcai.riken.jp/profile.cgi).

**Figure 5:**
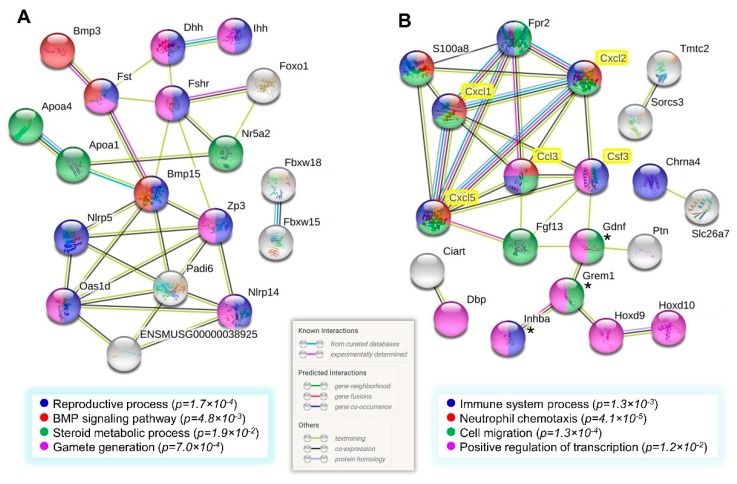
Protein–protein interaction network of genes associated with follicles and innate immunity in aged multiparous ovaries: The follicular-like (**A**) and the cytokine-like (**B**) gene clusters were analyzed with STRING v11 for known and predicted relationships existing as protein–protein interaction networks. The corresponding official *Mus musculus* gene symbols for the cytokines measured in serum were added to both the follicular (27) and the cytokine (32 and 6) clusters separately, to run each analysis. Serum cytokines are highlighted in yellow boxes in (**B**). The confidence score was set to 0.350, and the unconnected nodes were excluded from the network. Boxes at the bottom of each network indicate color codes for GO terms enriched in the respective protein networks (adj *p* < 0.05). (*) indicate genes that belong to the cytokine cluster, but show primary evidence of follicle function (see Table 2 and Table 3). Legend to color-coded interaction lines were adapted from the STRINGv11 output.

For this analysis, the transcriptome results were analyzed across the three experimental groups, (i.e., young, aged virgin and aged multiparous ovaries). The quality threshold clustering (QTC) algorithm was applied to the normalized expression levels of the 65 DEGs between the aged ovaries, and included the expression levels observed in young ovaries. This resulted in the following three clusters: 27 (Figure 2B), 32, and 6 genes (Figure 4B,D). Notably, the centroid pink colored line of the 27-genes cluster (Figure 2B, right) resembled the follicle count pattern depicted in Figure 2A (i.e., highest in young, lowest in aged virgin, and intermediate level in aged multiparous ovaries), and thereby it may be considered a “follicular-like” signature. Importantly, 11 from the 15 genes annotated in the GO terms related to oocyte–follicle homeostasis (Table 2), and 10 of the 11 genes with manually collected evidence of roles in oocyte–follicle homeostasis (Table 3) showed this follicular signature (Figure 2B). These 21 genes are shown in the intersection between “Follicle GO and literature” and “Follicle cluster” in the Venn diagram of Figure 2C. Interestingly, the intersection between “Follicle GO and literature” and “Cytokine cluster” captured five genes with a cytokine-like pattern, but functional evidence of follicle–oocyte involvement (Figure 4B, blue dot labels). These were *Inhba*, *Gdnf*, *Grem1*, *Hsd17b1*, and *Kcne2*. Finally, the genes *Apoa1*, *Gdnf*, *Grem1*, *Ihh*, *Inhba*, and *Zp3*, were common between the follicle/oocyte and immune/inflammatory themes based on GO. Similarly, *Inhba*, *Gdnf*, and *Grem1,* besides linking the follicle/oocyte and immune/inflammatory themes, exhibit a transcriptional modulation activity and take part in the cytokine-like gene regulatory network (Figure 5B).

### 3.6. Parity-Dependent Serum Cytokine Levels and Correlation with the Ovarian Transcriptome

Previously, we reported circulating levels of seven cytokines that increased with reproductive aging irrespective of parity records, and then decreased in response to tumor induction exclusively in multiparous compared with virgin mice [18]. Here, we show serum levels of other cytokines in intact (non-tumor induced) mice that, except CXCL1, remained unchanged between young and aged virgin mice, while increased significantly in aged multiparous animals. Figure 4A,C shows circulating levels of cytokines CXCL1 (GRO-alpha/KC), CXCL2 (MIP-2/GRO-beta), CXCL5 (LIX/ENA-78), CCL3 (MIP1-alpha), and CSF3 (G-CSF) in young, aged virgin, and aged multiparous mice. The aged multiparous mice showed the highest CCL3 and CSF3 levels. The [aged multiparous/aged virgin] mean ratio levels of these five cytokines ranged from 4.2-fold for CSF3 to 1.6-fold for CXCL2.

Interestingly, the cytokine serum levels were mirrored by the expression patterns of two clusters containing 32 (Figure 4B) and 6 (Figure 4D) significantly up-regulated genes in multiparous compared with virgin ovaries. This cytokine-like profile substantially differed from the above mentioned follicular-like pattern (27-genes), in that their mean gene expression levels in young ovaries were not drastically higher than those of the aged ovaries. Notably, the leukocyte chemoattractant genes *Fpr2* and *S100a8* were among the six-genes cluster showing the highest expression levels in the three conditions (Figure 4D).

The presence of immune cells in the ovary, with macrophages as the more abundant, is inherent to various physiological processes during fertile life, including follicle maturation, ovulation, luteogenesis/luteolysis, atresia, and vascular integrity [27,28,29]. As follicle depletion and ovarian aging progress, this immune cell content and their activities would become modulated by the SASP effect. Thus, the genes composing this cytokine-like pattern were mined in the Reference Database of Immune Cells (Ref-DIC) [30], with an emphasis on the neutrophil and macrophage gene expressions.

As shown in Figure 4E, *Fpr2*, *S100a8*, and *Serpina3g* are expressed in detectable levels in these two cell types. Both peritoneal and J774.1 (blood) macrophages show an increased *Fpr2* expression in response to a pro-inflammatory stimulus (LPS) at various times and doses, while the *Fpr2* expression by neutrophils is constitutively high. The *Serpina3g* expression was enhanced only in LPS-stimulated peritoneal macrophages and in the liver neutrophils from *Tcra* knockout mice. On the other hand, *S100a8* was weakly induced by LPS in macrophages, but highly expressed by the three neutrophil types shown without any stimulus. Regarding the cluster of 32 genes, Ref-DIC demonstrated high (*Eif4e3*, *Dtx4*) and moderate (*Cdk5rap2*, *Dbp*, *Actr3*) expression levels in the J7774.1 macrophages. Interestingly, *Ihnba* showed a high expression exclusively in peritoneal macrophages (data not shown). A recent report found that LPS treatment improved the innate immunity in a xenograft model of SKOV3 cells in CD-1 mice, resulting in extended survival and increased CXCL1 levels [31], one of the chemokines that was also detected here at higher levels in multiparous mice (Figure 4A).

### 3.7. Network Analysis of Gene Clusters of Higher Expression in the Multiparous Ovary

The genes comprising the follicle and cytokine clusters display some degree of overlap in enriched GO terms shown in Figure 2B and Table 2. Aimed to visually illustrate such an overlap and to uncover novel gene relationships, protein–protein interactions (PPI) networks were constructed with STRING v11.0 on both the 27 genes cluster referred to as the follicular-like signature, and the 38 (32 and 6) genes cluster corresponding to the cytokine-like signature defined in Section 3.4. The network of Figure 5A depicts *Fst*, *Fshr*, *Zp3*, *Bmp15*, *Oas1d*, and *Padi6* as major interconnected hubs among the descriptors at the bottom.

The networks shown in Figure 5 were obtained by adding the official gene symbols of the five cytokines measured in serum to each of the 27 or the 38 genes listed separately prior to running the STRING tool. Notably, only the network formed with the cytokine-like cluster (38 genes) showed significant known and predicted links between the proteins coded by those genes and the five cytokines. *S100a8* is indexed in three of the four GO terms at the bottom, and showed links with *Fpr2* and the three CXC cytokines only. In turn, all of the cytokines, except CSF3, were connected to *Fpr2* (Figure 5B). Precisely, these two genes overlap the majority of GO terms related to the immunity/chemotaxis component described in Section 3.2.

## 4. Discussion

The current knowledge on the gene expression by the aged mammalian ovary is scarce and limited to a debated steroidogenic capacity (Table 1). Epidemiology indicates that parity protects against OC, though the precise mechanism has not been elucidated. Smith and Xu speculated that the “depletion of germ cells and the loss of ovarian follicular function that follows might underlie the link between reproductive factors and ovarian cancer risk” [4]. The postulated protective role of the ovarian reserve against OC is supported by decreased OSE hyperplasia in the presence of a minimal number of follicles in ovaries of the white spotting variant (Wv) mice, a model of ovarian aging and menopause [8]. Thus, we addressed the relationship between parity and follicle depletion in the intact C57BL/6 mouse at estropause (menopause-like) age. Some of the mouse genes detected in the present study might be candidates to validate human postmenopausal ovaries according to parity history and their genetic variants investigated concerning OC risk and associated comorbidities in postmenopausal women.

### 4.1. Evidence of Remnant Follicles in the Aged Multiparous Ovary

Our results suggest that the aged multiparous mouse ovaries contain a higher follicle number and express higher transcript levels of several well-characterized follicle/oocyte related genes compared with the aged virgin ovary. Here, it follows that germ cell depletion is less, owing to pregnancy during prior fertile life. This finding agrees with results by Moini et al. who observed a higher ovarian reserve in parous women of reproductive age (mean 28 years old, range 20–35) measured as antral follicle counts and serum anti-müllerian hormone (AMH) [14]. Then, our data allow to reasonably speculate that this parity-dependent differential ovarian reserve might persist until an advanced age, thereby providing a protective action. In this regard, follicle depletion in women is not absolute at menopause. Approximately 1000 non-growing follicles remain in the human ovary at 51 years old, the mean menopause age [13], whereas the total non-growing follicle exhaustion in women is predicted to occur by 74 years old [32].

Among the follicle-/oocyte-related genes expressed by aged multiparous ovaries, seven were members of the TGF-β superfamily, which comprises over 30 conserved protein ligands classified in the following three major subfamilies: TGF-βs, activin/inhibins, and BMPs. These ligands are involved in processes such as embryonic development, tissue regeneration, wound healing, immunity, and reproduction. Ovarian TGF-β factors participate in somatic and germ cell growth and differentiation, ovulation, and fertilization, and play key roles in primordial follicle formation, assembly, and activation [33]. *Inhba* codes for the β-A subunit of inhibin-A, activin-A, and activin-AB dimers expressed by granulosa cells. Circulating inhibins repress, while activins stimulate pituitary FSH synthesis and release. Female reproductive aging is characterized by increased levels of circulating activin and FSH [34], the latter confirmed in our previous report [18]. Thus, a higher *Inhba* expression in the aged multiparous ovary suggests elevated levels of the activin-A homodimer. Intraovarian activins promote the autocrine expression of *Fshr* and *Cyp19a1* (aromatase) in pre-antral follicles, while depressing steroidogenesis in advanced-stage follicles and lutein-granulosa cells [34]. Consistently, we detected a higher *Fshr* expression in aged multiparous with respect to aged virgin ovaries. The coexpression of *Cyp19a1* showed the same trend, although not reaching statistical significance. In agreement with our findings, the FSH receptor (FSHR) and aromatase have been localized in the human postmenopausal ovary (Table 1) [35,36].

Other TGF-β related genes highly expressed by the multiparous ovary were the ligand antagonists *Fst* (follistatin) and *Grem1* (Gremlin-1) [33]. The proteins coded by these genes were detected in the stromal cells of human postmenopausal ovaries, and are proposed to neutralize the activities of endogenous follicular growth factors [37]. Although the parity status of the ovary samples analyzed by Jabara et al. was not specified [37], it could be assumed that parous postmenopausal ovaries were used. Follistatin is a high-affinity inhibitor of activin that blocks pituitary FSH release. However, as circulating follistatin levels decrease at menopause—in contrast to activins and FSH—the inhibition of systemic activin would not operate at this life stage [38]. Ovarian *Fst* is expressed as three isoforms that regulate the balance between germ-cell nest breakdown and the subsequent assembly of primordial follicles. Mouse *Fst* overexpression blocks follicle maturation before the antral stage, possibly by inhibiting granulosa cell proliferation [39], a role relevant in the aged multiparous ovary containing residual follicles as our data suggest.

Finally, the oocyte-specific growth factor *Bmp15* was another TGF-β/BMP transcript showing a parity-dependent differential expression. This BMP is detectable at the primordial stage and regulates the ovulatory rate by preventing the premature activation of primordial follicles [40]. In addition, FSH, which is increased during menopause (and estropause) and signals via *Fshr*, also restricts activation of primordial follicles as observed in fertility studies [41,42] thus preventing depletion of the resting ovarian follicle pool. Consistent with the above-described preventive roles, *Bmp15*, *Fshr,* and *Fst* are interconnected in the protein network (Figure 5A). The function of TGF-β/BMP antagonists BMP15 and activin-A in sustaining *Fshr* expression, suggest the existence of a higher dormant follicle reserve in the aged multiparous compared with the aged virgin ovary. Follicle count results (Figure 2B) support this idea. Interestingly, the second TGF-beta antagonist detected in this study, *Grem1*, displayed a cytokine-like pattern (Figure 5B).

### 4.2. Former Parity Might Sustain Residual Steroidogenesis in the Aged Ovaries

The research on post-reproductive ovaries has mostly dealt with its controversial androgenic capacity (Table 1), which would rely on the accumulation of secondary interstitial cells derived from theca cells of atretic follicles [43]. Here, we provide evidence that this ability might be based on a former parity history. As shown in Table 2, six steroidogenic genes were highly expressed in the aged multiparous ovary. *Hsd17b1* codes for the type-1, 17-beta hydroxysteroid dehydrogenase, which catalyzes the NADPH-dependent reduction of estrone and androstenedione to estradiol and testosterone, respectively. Given that estrone is produced by aromatase, our data suggest a synthesis of testosterone only, if the HSD17B1 enzyme levels resemble the transcript levels in the multiparous mouse ovary. This conclusion is supported by the presence of testosterone; its precursor androstenedione [44,45]; and the HSD17B1 protein in the OSE, cysts, stroma, and vascular endothelium of human post-menopausal ovaries [36]. Interestingly, given that follicle viability is improved by androgens such as dehydroepiandrosterone (DHEA) in an ovarian aged rat model [46], our data suggest that the steroidogenic capacity of aged ovaries detected in the present and other studies contributes to preserve follicle remnants. Like follicle/oocyte transcripts, steroid synthesis, including androgen production by the human postmenopausal ovary, has not been linked to parity history yet. None of the age-related ovarian gene expression reports compiled in Table 1 specify parity records. Then, it seems plausible to think that the residual steroidogenic capacity detected in the aged human postmenopausal ovary in such studies might be derived from follicle remnants persisting in parous ovaries because of pregnancy during former fertile age.

### 4.3. Transcriptional Control in the Aged Multiparous Ovary

Six genes up-regulated in aged multiparous ovaries display an activity of transcription factor. In general, age-dependent up-regulation involves stress-responsive transcription factors. The forkhead box (FOXO) family of transcription factors is implicated in longevity and aging-related processes, including cell cycle arrest, oxidative stress, DNA repair, apoptosis, and autophagy [47]. *Foxo1* is a master regulator of FSH-target genes in granulosa cells [48]. It is highly expressed in growing follicles, repressed during luteinization, and induced in atretic and developing cystic follicles of middle-age acyclic rats at persistent estrous [49]. During fertile age, the apoptotic and autophagic actions of *Foxo1* on granulosa cells are associated with oxidative stress and can be inhibited by FSH in mice [50,51]. In the presence of activins, FSH induces AKT-mediated *Foxo1* phosphorylation, thereby releasing the nuclear transcriptional repression of steroid and follicle-related genes such as *Fshr* and *Nr5a2,* among others [52]. In turn, *Nr5a2* is a zinc finger transcription factor (orphan nuclear hormone receptor) essential for luteinization [53]. Thus, our findings on *Foxo1*, *Fshr,* and *Nr5a2* co-expression under persistent FSH stimulation in the presence of activin-A (*Inhba*), suggest phosphorylated *Foxo1* in the aged multiparous ovary. This conclusion is reflected in the follicular-like protein network shown in Figure 5A. If this transcriptional pattern is preserved at a protein level, we hypothesize that a direct interaction of *Foxo1* with membrane-localized *Fshr* to form an atypical signaling complex would preclude the nuclear translocation of *Foxo1* in aged multiparous ovarian cells, as observed in HEK 293 cells [54].

Also, the Notch target *Hey2* codes for a myc-type basic helix–loop–helix transcription factor highly expressed in the adult mouse ovary (https://www.ncbi.nlm.nih.gov/gene/15214#gene-expression). *Hey2* connects TGF-β and Notch pathways during proliferation of pre-antral granulosa cells [55] and modulates the homeostasis of the somatic-germ cell syncytia during follicular development [56]. Importantly, Notch signaling has been recently associated with innate immunity and the onset of inflammatory, age-related diseases [57].

### 4.4. Parity History Improves the Ovarian and Systemic Immune-Chemotactic Activity of Aged Mice

Ovarian processes, including ovulation, luteinization, and atresia, depend on a delicate pro-/anti-inflammatory balance relying on leukocyte activities [27,28,29,58]. Macrophages are the most abundant ovarian leukocytes, and their function in the ovary and other sections of the female reproductive system during fertile age is modulated by estrogens to induce the alternative, M2 anti-inflammatory, tissue repair differentiation profile [29]. The available information about ovarian immunity and inflammation during the postmenopausal phase and parity history is scarce.

Higher circulating levels of CXCL1, CXCL2, CXCL5, CSF3, and CCL3 were detected in aged multiparous mice. This cytokine set participates in the innate immune response to endogenous tissue damage and infection mainly in initial phases [59]. A role in leukocyte extravasation to inflammatory lesions is suggested by the co-localization of CXCL1, CXCL2, and CXCL5 in granules of a subset of vessels in healthy mouse skin and platelets [60]. Fertile-age human granulosa-lutein cells express high levels of CXCL2 and other cytokines after prostaglandin F2-alpha treatment or the removal of luteotropic stimulants, suggesting a role in luteolysis [61]. The neutrophil-recruiting abilities of these chemokines depend on activation and binding to their CXCR1 and CXCR2 receptors [59]. Consistently, in our results, the *Cxcr1* gene was upregulated almost six-fold (adj-*p* 2.96 × 10^−5^) in aged with respect to young ovaries, regardless of parity status (not shown).

The local CXCL1 and CXCL2 synthesis decrease with age, thus promoting infection spread in a mouse model of soft-tissue and skin infection [62]. Age-exacerbated bacterial growth and delayed wound healing in experimental infection have been successfully treated with exogenous CSF3 to enhance neutrophil recruitment [63]. As aging impairs neutrophil and macrophage chemotaxis further than phagocytic or bactericidal capacities [64], our results suggest that increased systemic levels of chemokines measured in aged multiparous mice might help to counteract such an age-dependent impairment of innate immune cell chemotaxis, thus improving endogenous tissue repair. Moreover, the local overexpression of the migration and chemotactic genes *Fpr2*, *S100a8*, *Sema5a*, *Fgf13*, *Gdnf,* and *Grem1* (Table 2, Figure 5B) in the aged multiparous ovary would reflect its repertoire of resident leukocyte cells, an idea supported by the analysis of Figure 4E, particularly regarding *Fpr2* and *S100a8*.

Neutrophils, monocytes, and activated macrophages express high levels of S100-calgranulin A (*S100a8*). This Ca-/Zn-binding protein forms homo- and hetero-dimers (with *S100a9*), which stimulate the migration of neutrophils to inflammatory sites [65] via tight binding to the CD68 antigen in LPS stimulated macrophages [66]. Interestingly, we found a high *Cd68* expression in aged relative to young ovaries, irrespective of parity status (3.9 fold; adj *p* = 7.8 × 10^−5^). Furthermore, the S100A8/9 heterodimer (calprotectin) is abundant in neutrophils, increases with age in the majority of mouse and human tissues [67], and displays growth-inhibitory and apoptosis-inducing activities on tumor cells and fibroblasts [68]. We observed an increasing trend of the S100a9 transcript level in aged multiparous ovaries, although not reaching statistical significance (raw *p =* 8.4 × 10^−3^). Neutrophil recruitment by S100A9 requires interaction with TLR2 and CXCL2 [69]. Consistently, the serum CXCL2 levels were increased in the aged multiparous relative to aged virgin mice (Figure 4A), while our microarray data indicated a *Tlr2* overexpression in aged relative to young ovaries (2.3 fold, adj-*p =* 4 × 10^−6^).

*Fpr2* codes for a member of the G-protein coupled formyl peptide receptor family that promotes the chemotaxis of immune cells to damaged tissue, thereby preventing inflammation-associated tumorigenesis [70]. *Fpr2* chemoattracts neutrophils and monocytes through chemokines CCL3 and CXCL8 [71]. Consistently, the serum CCL3 levels were higher in the aged multiparous mice in the present study (see Section 3.6). The chemotactic and tissue repair abilities of *Fpr2* rely on its ability to sense a wide range of danger (and pathogen)-associated molecular patterns (DAMPs), allowing for neutrophil activation and the subsequent resolution of inflammation [72].

### 4.5. Apoptotic Activity in the Aged Multiparous Mouse Ovary

In aged tissues, apoptosis and senescence may play complementary roles as tumor-suppressor mechanisms [73]. Thus, the positive regulation of apoptosis in the reproductively aged multiparous ovary might act as a safeguard mechanism against pre-neoplasia initiation in a chronic-inflammatory senescent tissue environment by the perpetuation of the SASP. We speculate that the genes associated with cell death (Figure 3B and Table 2) might be involved in the apoptosis of the interstitial cells of a follicular origin, thereby facilitating the action of macrophages. Accordingly, the human early postmenopausal ovaries show apoptotic markers that decay from early to late post-menopause [74], although a parity link has not been suggested yet. During the fertile cycle, cell death is relevant in atresia and luteolysis.

Apoptosis might be also related to the roles of the resident leukocytes of the aged ovary (see Section 3.6). The SASP can lead to chronic deleterious effects because of age-impaired immune function (immune-senescence). Therefore, cell immunity genes, particularly those associated with leukocyte chemotaxis, would promote an efficient removal and processing of apoptotic cells in the aged multiparous ovary. Neutrophil apoptosis is required to activate the phagocytic capacity of macrophages, thereby resolving inflammation [75]. In contrast, this process would be impaired in the aged virgin ovary, thus leading to an excessive accumulation of incompletely digested cell debris in the form of lipofuscin, as we recently reported [22]. Ovarian lipofuscin might be the consequence of an overload of atretic material during reproductive aging, which is not properly cleared, thus accumulating because lysosomal and/or proteasomal dysfunction by age-induced cell damage, as has been described in other tissues [76].

## 5. Conclusions

Here, we applied an integrative analysis (GO, GSEA, PPI networks, and Ref-DIC) to the ovarian transcriptome and systemic chemokine profiles observed in the naturally aged C57BL/6 mouse ovary so as to gain insight into the molecular basis of OC risk reduction by parity history. The mice were maintained in multiparous versus virgin regimens until estropausal age, equivalent to early post-menopause in women. Regardless of parity status, the aged ovary expressed a high content of genes involved in immune/inflammatory response, cell adhesion, TNF synthesis, proteolysis, and wound healing, thus reflecting a low-grade chronic inflammation due to endogenous tissue damage, which is characteristic of SASP. Leukocyte markers, including macrophage, neutrophil, lymphocyte, and mast cell transcripts, were also detected in the aged ovary. More importantly, aged multiparous ovaries showed a higher residual content of primordial and total follicles, relative to aged virgin ovaries. This finding agreed with the higher transcript levels of the 26 DEGs—out of the 65 total—with reported evidence of the roles in the homeostasis of oocytes, follicles, and the ovary, particularly genes preventing the exhaustion of the primordial follicle pool. The remaining DEGs were enriched in innate immunity mainly related to neutrophil and macrophage chemotaxis, while their expression profile resembled the circulating levels of the CXCL1, CXCL2, CXCL5, CSF3, and CCL3 chemokines implicated in the repair of endogenous tissue damage. We conclude that the aged multiparous ovary retains a remnant follicle reserve that evades either maturation or atresia, probably due to the combined action of paracrine *Tgf/Bmp* antagonists and the modulation of *Fshr-Foxo1* signaling. Coincident with this residual follicular reserve, past parity would enhance the immune-surveillance capacity of resident ovarian macrophages and neutrophils by improving their phagocytic ability on apoptotic atretic cells, thereby minimizing the deleterious effects of SASP. In summary, the observed protection of pregnancy (or progestin-induced anovulation) against ovarian neoplasia may be regarded as a long-term “imprint”, leading to persistent remnant follicles with concomitant robust innate immunity in the aged multiparous ovary. How this imprint is generated during a woman’s fertile life and overcomes the effects of ovarian aging it remains to be investigated.

## Figures and Tables

**Table 1:** Previous knowledge on gene expression in the human post-menopausal ovary.

Gene (alias)	Context (PMIDs) ^a^	Ovarian Expression (This Work)
*FST* (follistatin)	Residual steroidogenesis; stroma (12519894)	Yes ^b^; ↑ multiparous
*GREM1* (gremlin−1)	Residual steroidogenesis; stroma (12519894)	Yes; ↑ multiparous
*LHCGR* (LH receptor)	Detected in OSE, cysts and stroma (22207559, 16253961)	No ^c^; ↑ aged
*FSHR* (FSH receptor)	Detected in OSE, cysts and stroma (22207559)	Yes; ↑ multiparous
*HSD3B* (3β-hydroxysteroid dehydrogenase)	Detected in the cortical stroma (2643063)	NDE ^d^
*PR* (progesterone receptor)	Detected in OSE, cysts and stroma (19056530, 20552551).	NDE
*ESR1* (estrogen receptor alpha)	Detected in OSE, cysts and stroma (18165170, 20552551).	NDE
*CYP19A1* (aromatase)	Detected in OSE, cysts, stroma and vascular endothelium (24855493)	NDE
*HSD17B1* (17β-hydroxysteroid dehydrogenase)	Detected in OSE, cysts, stroma and vascular endothelium (24855493)	Yes, ↑ multiparous
*AR* (androgen receptor)	Detected in the OSE, cysts and stroma (20552551)	NDE
*APCS* (serum amyloid P component)	Differential pre-/post-menopausal proteome (25037597)	No; ↑ young (*)
*HSPB1* (Hsp27)	Differential pre-/post-menopausal proteome (25037597)	NDE
*GLO1 (glyoxalase-I)*	Differential pre-/post-menopausal proteome (25037597)	NDE
*UCHL1* (ubiquitin C-terminal hydrolase L1)	Differential pre-/post-menopausal proteome (25037597)	No; ↑ young

^a^ PMID stands for PubMed unique identifier. ^b^ “Yes” indicates a gene appearing in the 177 DEGs according to parity history (see Section 3.2). Arrow indicates predominant multiparous or nulliparous expression. ^c^ “No” indicates that expression was not parity-dependent, but only part of the 904 DEGs according to age (see Section 3.2). Arrow indicates predominant young versus aged expression. ^d^ NDE: Non differentially expressed gene in either the aged-young or the aged virgin-aged multiparous comparisons. * Differential expression with *p* < 0.05 but under the fold change threshold. OSE—ovarian surface epithelium.

**Table 2:** Enriched GO terms in genes of higher expression in aged multiparous ovaries ^a^.

Functional Theme	GO Terms Associated	Genes
Follicle and oocyte homeostasis	Ovarian follicle development Germ cell development TGF-beta receptor binding BMP signaling Steroid metabolic process	*Bmp15*, *Fshr*, *Inhba*, *Oas1d, Dhh, Ihh, Zp3*, *Gdnf*, *Bmp3*, *Fst*, *Grem1*, *Hsd17b1*, *Apoa1*, *Apoa4*, and *Nr5a2*
Cell immunity and inflammation	Regulation of inflammatory response Positive regulation of immune system process Chemotaxis Cell migration	*Apoa1*, *S100a8*, *Zp3*, *Fpr2*, *Ihh*, *Inhba*, *Masp1*, *Rftn2*, *Sema5a*, *Fgf13*, *Gdnf*, *Grem1*, and *Efna5*
Transcription	Positive regulation of transcription, DNA-templated Transcription factor activity, sequence-specific DNA binding	*Bmp15, Bmp3, Cdk5rap2, Dbp, Foxo1, Gdnf, Grem1, Hey2, Hoxd10, Hoxd9, Ihh, Inhba, Nr5a2,* and *Zp3*
Cell death	Cell death Positive regulation of apoptotic process	*Nlrp5*, *S100a8, Serpina3g*, *Foxo1*, *Inhba*, and *Ptn*

^a^ GO analysis was done with VLAD. Enrichment was more than two-fold with adj *p* < 0.05. The analysis covered 31 of the 65 DEGs of a higher level in aged multiparous versus aged virgin ovaries. BMP—bone morphogenetic protein.

**Table 3:** Follicle and oocyte-related genes not annotated in GO ^a^.

Gene	Fold Change ^b^	Role in Ovarian Biology	PMID
*Nlrp14*	6.6	Innate immunity of oocytes	28423339
*Slc38a5*	5.2	Paralog *Slc38a3* is expressed in adult mouse granulosa cells	23083410
*Padi6*	5.1	Part of cytoplasmic lattice and cytoskeletal sheets in oocytes	17587491, 27929740
*Nlrp5*	4.6	ER, calcium and mitochondrial homeostasis of oocytes	24374158, 22357545
*Cpsf4l*	2.9	Expressed in adult mouse granulosa cells	23083410
*E330034G19Rik*	2.8	Oocyte specific in mouse; meiotic maturation	17567914
*Hey2*	2.7	Notch target involved in oocyte-follicle growth balance	24552588
*Fbxw15*	2.4	Oocyte specific. Follicle assembly and early follicle growth	18094359
*Serpine2*	2.2	Cumulus cell marker of oocyte maturation	23082142
*Efna5*	2.0	Regulates proliferation and apoptosis of granulosa cells	29619874
*Kcne2*	1.9	Expressed in adult mouse granulosa cells	23083410

^a^ Genes in this table are part of the 27 genes with a follicular-like expression pattern (highest level in young, lowest in aged virgin, and intermediate in aged multiparous ovaries), as shown in Figure 2C. ^b^ The fold change here corresponds to the [aged-multiparous/aged virgin] expression ratios in the direct linear scale.

## Supplementary Materials

The following are available online at https://www.mdpi.com/2218-273X/10/1/113/s1. Supplementary File 1: Excel spreadsheet containing the list of 177 DEGs with log_2_ FC results and gene identifiers. Supplementary File 2: Methods in detail.
