# Supplementary material for: The Ovarian Transcriptome of Reproductively Aged Multiparous Mice: Candidate Genes for Ovarian Cancer Protection
Source: Biomolecules. 2020 Jan 9;10(1):113. doi: 10.3390/biom10010113 (PMC7022285; doi:10.3390/biom10010113)
Supplement: Supplementary file 1 [file biomolecules-10-00113-s001.zip › biomolecules-635007-Suppl-file-2.docx]

*Methods in detail (Urzua et al; Biomolecules-635007)*

Subheadings from the Methods section of main text were conserved.

1. Microarray Profiling of Mouse Ovaries

RNAs were amplified with the Ambion Illumina RNA amplification kit (Ambion, Austin) to generate a biotinylated cRNA. Briefly, 550 ng of total RNA were reverse-transcribed with a T7 oligo(dT) primer. After second-strand cDNA synthesis, in vitro transcription was performed with T7 polymerase plus biotin-labeled NTPs. This labeled cRNA was purified and quantified; 750 ng of labeled cRNA were hybridized to Mouse Ref-8 expression v.2 bead arrays for 16-18 h at 58°C, according to manufacturer's instructions (Illumina, Inc., San Diego, USA). The addition of fluorolink streptavidin-Cy3 (Amersham, GE Healthcare Bio-Sciences, UK) allowed detection of array signal with an Illumina confocal scanner. The Illumina Mouse Ref-8 expression v.2 bead-arrays contain 25,697 distinct oligonucleotide probes to cover 17,640 unique mouse coding genes.

2. Determination of serum chemokines

Twenty-five μL of serum were diluted with 25 μL of Assay Buffer and loaded to the plate in addition to control and standard samples provided in the kit. Then, 25 μL of the Pre-Mixed Beads mixture were added to each well, plate sealed, covered with foil and incubated overnight with shaking at 4°C. The entire content of the plate was gently discarded, washed twice, and 50 μL of Detection Antibodies solution were added and incubated for 1 hr at room temperature. Then, 50 μL of Streptavidin-Phycoerythrin solution were added and incubated further 30 min at room temperature. Plate content was gently discarded and washed twice. Finally, 100-150 μL of Sheath Fluid were added to each well and beads were resuspended by shaking for 5 min. Median fluorescence intensity of beads was read in a Luminex 200™ instrument (Luminex Corp., USA) operated through the xPONENT 3.1 software. Data processing was conducted with the Milliplex Analyst 3.5.5.0 software (Merck, USA) using the 5-parameter logistic curve.

3. Quantitative RT-PCR primer sequences

Amplification cycles were: Step 1: 95°C for 10 min, step 2: 95ºC for 20 secs, step 3: Tm of each primer pair for 20 secs, step 4: 72ºC for 20 secs. Steps 2 to 4 were repeated 45 times. After the last cycle, a melting curve was obtained between 70-95ºC at 0.5ºC intervals. Real-time fluorescence data were captured and processed with the RotorGene-6000 software v1.7 (Qiagen, GmBH). Quantification of mRNAs was based on Ct values, which is defined as the PCR cycle at which an increase in reporter fluorescence above the baseline signal can be detected. Normalization was done with the Rn18s transcript assayed in both the test and the reference RNA samples [16]. The delta Ct-sample value (Ct-sample =Ct sample - Ct reference) was transformed by taking the result of the expression 2(-Ct) - 1 > 0 then the result = 2(-Ct) - 1 or else the result =-1/2(-Ct). This calculation converted the linear range ratio for down-regulation from (0.0)-(1.0) to (-∞)-(0.0) and for up-regulation from (1.0)-(+∞) to (0.0)-(+∞) in the log2 scale. Gene symbol and forward/reverse sequences and Tms were as follows:

*Foxo1*, 5´-CTGCTCAATGCCACTGAATG-3´/5´-CAACTGCCCAT GATTCACAC-3´; (60°C/60°C)

*Fst*, 5´-GTAATCGGATTTGCCCAGAG- 3´/5´-CCGCCACACTGGATATCTTC-3´; (60°C/62°C)

*Fshr*, 5´-CCTTGCTCCTG GTCTCCTTG-3´/5´-CTCGGTCACCTTGCTATCTTG-3´; (64°C/64°C)

*Alas2*, 5'-CCCCTCCAAGATGTGTCTGT-3'/5'-CATATTGGGGTCCCATGTTC-3'; (62°C/60°C)

*Snca*, 5'-AGGCAGCTGGAAAGACAAAA-3'/5'-CAGCTCC CTCCACTGTCTTC-3'; (58°C/64°C)

*Hba-a1*, 5'-CTGGGGAAGACAAAAGCAAC-3'/5'-GCCGTGGCTTACATCA AAGT-3', (60°C/60°C)

*Hbb-bt*, 5'-ATGGCCTGAATCACTTGGAC-3'/5'-ACGATCATATTGCCCAGGAG-3'; (62°C/62°C)

*Rn18s*, AAACGGCTACCACATCCAAG-3'/5'-CCTCCAATGGATC CTCGTTA-3' (59°C/59°C)
